# Interim estimates of 2016/17 vaccine effectiveness against influenza A(H3N2), Canada, January 2017

**DOI:** 10.2807/1560-7917.ES.2017.22.6.30460

**Published:** 2017-02-09

**Authors:** Danuta M Skowronski, Catharine Chambers, Suzana Sabaiduc, James A Dickinson, Anne-Luise Winter, Gaston De Serres, Steven J Drews, Agatha Jassem, Jonathan B Gubbay, Hugues Charest, Robert Balshaw, Nathalie Bastien, Yan Li, Mel Krajden

**Affiliations:** 1 British Columbia Centre for Disease Control, Vancouver, Canada; 2 University of British Columbia, Vancouver, Canada; 3 University of Calgary, Calgary, Canada; 4 Public Health Ontario, Toronto, Canada; 5 Institut National de Santé Publique du Québec (National Institute of Health of Quebec), Québec, Canada; 6 Laval University, Quebec, Canada; 7 Centre Hospitalier Universitaire de Québec (University Hospital Centre of Quebec), Québec, Canada; 8 Alberta Provincial Laboratory, Edmonton, Canada; 9 University of Alberta, Edmonton, Canada; 10 University of Toronto, Toronto, Canada; 11 National Microbiology Laboratory, Public Health Agency of Canada, Winnipeg, Canada

**Keywords:** influenza, influenza virus, influenza-like illness - ILI, vaccine-preventable diseases, vaccines and immunisation, effectiveness

## Abstract

Using a test-negative design, the Canadian Sentinel Practitioner Surveillance Network (SPSN) assessed interim 2016/17 influenza vaccine effectiveness (VE) against dominant influenza A(H3N2) viruses considered antigenically matched to the clade 3C.2a vaccine strain. Sequence analysis revealed substantial heterogeneity in emerging 3C.2a1 variants by province and over time. Adjusted VE was 42% (95% confidence interval: 18–59%) overall, with variation by province. Interim virological and VE findings reported here warrant further investigation to inform potential vaccine reformulation.

The 2016/17 season in Canada has been characterised by dominant influenza A(H3N2) activity, increasing since late November 2016 but with regional variation in timing and intensity from west to east [1]. We assessed interim 2016/17 vaccine effectiveness (VE) against influenza A(H3N2) viruses collected through the Canadian Sentinel Practitioner Surveillance Network (SPSN). Detailed genetic characterisation of sentinel viruses was undertaken to assess the contribution of emerging clade 3C.2a1 variants and their potential impact on protection conferred by the clade 3C.2a vaccine, specifically the A/Hong Kong/4801/2014(H3N2)-like component.

## Virological and vaccine effectiveness evaluation 

As previously described [2,3], nasal/nasopharyngeal specimens collected from patients aged 1 year and older presenting within 7 days of influenza-like illness (ILI) onset to community-based sentinel practitioners in four provinces (Alberta, British Columbia, Ontario and Quebec) were included in the interim analysis. Epidemiological information was collected at the time of specimen collection using a standard questionnaire. Ethics review boards in each province approved the study.

**Figure 1:**
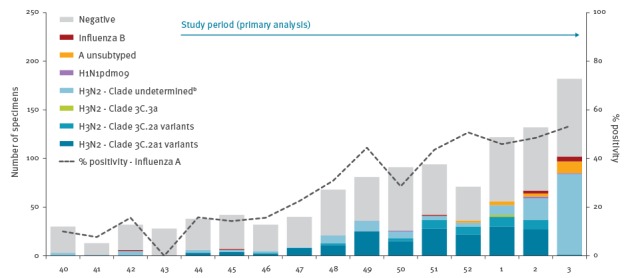
Influenza detections by type/subtype/clade and week of specimen collection, Canadian Sentinel Practitioner Surveillance Network, 2 October 2016–21 January 2017 (n = 1,096)^a^

^a^ Specimens were included if the patient: (i) met the influenza-like illness case definition (requiring fever and cough and at least one or more of sore throat, arthralgia, myalgia or prostration; fever was not required in elderly patients ≥ 65 years-old), (ii) had specimen collection ≤ 7 days after symptom onset, (iii) was ≥ 1-year-old at the time of onset, and (iv) had valid laboratory RT-PCR results. Specimens are displayed in the epidemic curve regardless of the patient’s vaccination status or timing of vaccination. Missing specimen collection dates were imputed as the date the specimen was received and processed at the provincial laboratory minus two days, the average time between specimen collection date and laboratory received date among specimens with complete information for both values.

^b^ Specimens with undetermined clade included those where sequencing was attempted but failed (n = 42) or sequencing was not attempted, e.g. those with insufficient viral load (n = 8), submitted after the start of the mid-season analysis on 21 January 2017 (n = 99), or excluded from primary vaccine effectiveness (VE) analysis (n=23).

Specimens collected between 1 November 2016 (week 44) and 21 January 2017 (week 3) were included in primary VE analysis, corresponding to the period during which influenza test positivity consistently exceeded 10% (Figure 1).

Influenza virus testing and influenza A subtyping were conducted using real-time RT-PCR assays validated for use at provincial reference laboratories, including in-house assays in Alberta [4] and British Columbia [5] and commercial assays in Ontario [6] and Quebec [7]. Sequencing of the haemagglutinin (HA) gene was attempted directly on all influenza A(H3N2)-positive patient specimens contributing to VE analysis that had sufficient viral load and that were available up to 21 January 2017 in order to determine clade designation and to identify mutations in established antigenic sites labelled A–E for H3N2 viruses [8,9].

VE was derived using a test-negative design [2,3]. Patients testing positive for influenza A(H3N2) were considered cases; those testing negative were considered controls. Patients who self-reported receiving at least one dose of influenza vaccine at least 2 weeks before ILI onset were considered vaccinated; those vaccinated less than 2 weeks before onset or who had unknown vaccination status or timing were excluded. Patients who did not meet the ILI case definition, those with specimen collection more than 7 days since ILI onset or ILI onset date unknown and those with indeterminate RT-PCR results were also excluded. Odds ratios (OR) were estimated using a logistic regression model, adjusted for age group, province, time from onset to specimen collection and specimen collection date (grouped into 2-week intervals). VE was derived as (1–OR) × 100%, comparing influenza A(H3N2) test positivity between vaccinated and unvaccinated participants. 

## Virological and vaccine effectiveness findings

A total of 932 specimens met study inclusion criteria. Influenza viruses were detected in 396 (42%) specimens, including 387 (98%) influenza A and nine (2%) influenza B. Of the 374 (97%) influenza A viruses with available subtype information, almost all (n = 370; 99%) were A(H3N2); four A(H1N1)pdm09 viruses were detected. VE analyses are presented for A(H3N2) only, including 370 test-positive cases and 536 test-negative controls (n = 906 overall). Working-age adults 20–64-years-old comprised the majority (57%) of the study sample (Table 1).

**Table 1:** Participant characteristics, interim vaccine effectiveness evaluation, Canadian Sentinel Practitioner Surveillance Network, 1 November 2016–21 January 2017 (n = 906)

Characteristic	Overall % (column)		Distribution by case status % (column)					Vaccinated % (row)						
			H3N2 cases		Negative controls		p value^a^	Overall		p value^a^	H3N2 cases		Negative controls	
	n	%	n	%	n	%		n	%		n	%	n	%
n % (row)	906	100	370	41	536	59	NA	246	27	NA	87	24	159	30
Age group (years)														
1–8	137	15	51	14	86	16	0.19	24	18	< 0.01	8	16	16	19
9–19	133	15	66	18	67	13		18	14		8	12	10	15
20–49	359	40	141	38	218	41		74	21		26	18	48	22
50–64	155	17	59	16	96	18		54	35		17	29	37	39
≥ 65	122	13	53	14	69	13		76	62		28	53	48	70
Median (range)	34 (1–97)		34 (1–91)		35 (1–97)		0.99	52.5 (1–97)		< 0.01	50 (1–90)		53 (1–97)	
Sex														
Female	524	58	205	56	319	60	0.20	154	29	0.09	44	21	110	34
Male	378	42	164	44	214	40		92	24		43	26	49	23
Unknown	4	NA	1	NA	3	NA	NA	0	NA	NA	0	NA	0	NA
Co-morbidity^b^														
No	664	80	270	81	394	79	0.52	147	22	< 0.01	49	18	98	25
Yes	166	20	63	19	103	21		77	46		28	44	49	48
Unknown	76	NA	37	NA	39	NA	NA	22	NA	NA	10	NA	12	NA
Province														
Alberta	278	31	110	30	168	31	0.03	71	26	< 0.01	20	18	51	30
British Columbia	327	36	134	36	193	36		92	28		37	28	55	29
Ontario	179	20	87	24	92	17		64	36		25	29	39	42
Quebec	122	13	39	11	83	15		19	16		5	13	14	17
Specimen collection interval from ILI onset (days)^c^														
≤ 4	687	76	316	85	371	69	< 0.01	174	25	0.03	70	22	104	28
5–7	219	24	54	15	165	31		72	33		17	31	55	33
Median (range)	3 (0–7)		3 (0–7)		3 (0–7)		< 0.01	3 (0–7)		0.03	3 (0–7)		3 (0–7)	
Specimen collection date (2-week interval)														
Weeks 44–45	64	7	10	3	54	10	< 0.01	4	6	< 0.01	0	0	4	7
Weeks 46–47	61	7	13	4	48	9		12	20		3	23	9	19
Weeks 48–49	139	15	54	15	85	16		31	22		12	22	19	22
Weeks 50–51	174	19	65	18	109	20		51	29		11	17	40	37
Weeks 52–1	184	20	86	23	98	18		58	32		24	28	34	35
Weeks 2–3	284	31	142	38	142	26		90	32		37	26	53	37

ILI: influenza-like illness; NA: not applicable.

^a^ Differences between cases and controls and vaccinated and unvaccinated participants were compared using the chi-squared test or Wilcoxon rank-sum test.

^b^ Includes chronic co-morbidities that place individuals at higher risk of serious complications from influenza as defined by Canada’s National Advisory Committee on Immunization (NACI), including: heart, pulmonary (including asthma), renal, metabolic (such as diabetes), blood, cancer, or immunocompromising conditions, conditions that compromise management of respiratory secretions and increase risk of aspiration, or morbid obesity (body mass index ≥ 40).

^c^ Missing specimen collection dates were imputed as the date the specimen was received and processed at the provincial laboratory minus two days, the average time between specimen collection date and laboratory received date among specimens with complete information for both values. Specimen collection interval was derived based on the number of days between ILI onset and the specified or imputed specimen collection date.

Overall 24% of cases and 30% of controls were considered vaccinated (p=0.04), corresponding to an unadjusted VE of 27% (95% confidence interval (CI): 1–46) against medically attended influenza A(H3N2) illness (Table 2). After adjustment for relevant covariates, VE was 42% (95% CI: 18–59).

**Table 2:** Interim vaccine effectiveness estimates for influenza A(H3N2), Canadian Sentinel Practitioner Surveillance Network, 1 November 2016–21 January 2017 (n = 906)

Model	n total	Cases		Controls		VE % (95% CI)
		n	% vaccinated	n	% vaccinated	
**Primary analysis^a^**						
Unadjusted	906	370	24	536	30	27 (1 to 46)
***Individual covariate adjustment ***						
Age group (1–8, 9–19, 20–49, 50–64, ≥ 65 years)						30 (4 to 50)
Province^b^						32 (7 to 50)
Specimen collection interval from ILI onset (≤ 4, 5–7 days)						23 (−5 to 44)
Specimen collection date (2-week interval)						38 (15 to 55)
***Full covariate adjustment ***						
Adjusted						42 (18 to 59)
**Restricted by province^c^**						
***Alberta***						
Unadjusted	278	110	18	168	30	49 (8 to 72)
Adjusted						62 (26 to 80)
***British Columbia***						
Unadjusted	327	134	28	193	29	4 (−56 to 41)
Adjusted						28 (−30 to 60)
***Ontario^d^***						
Unadjusted	179	87	29	92	42	45 (−2 to 71)
Adjusted						27 (−60 to 66)
***Quebec***						
Unadjusted	122	39	13	83	17	28 (−118 to 76)
Adjusted						NE
***All provinces excluding Alberta***						
Unadjusted	628	260	26	368	29	16 (−19 to 42)
Adjusted^e^						34 (−1 to 57)

CI: confidence interval; ILI: influenza-like illness; NE: not estimated (insufficient sample size); VE: vaccine effectiveness.

^a^ Analysis adjusted for age group, province, specimen collection interval from ILI onset and specimen collection date (2-week interval).

^b^ Alberta, British Columbia, Ontario, Quebec.

^c^ Analysis adjusted for age group, specimen collection interval and specimen collection date (2-week interval).

^d^ Due to logistical issues, specimen collection for the 2016/17 season began late in Ontario. The study period for Ontario-specific VE analysis was defined as 12 December 2016 (week 50) to 21 January 2017 (week 3).

^e^ Analysis adjusted for age group, province (British Columbia, Ontario, Quebec), specimen collection interval and specimen collection date (2-week interval).

Genetic clade information was available for 221 of 263 (84%) influenza A(H3N2) sentinel specimens for which sequencing was attempted. The majority of viruses (176/221; 80%) clustered with the newly emerging clade 3C.2a1, defined by N171K +/− N121K mutations in site D, with most (165/176; 94%) having between one and three additional antigenic site mutations (Table 3). Other clade 3C.2a variants, each with two or three antigenic site mutations, comprised 43 (19%) sequenced influenza A(H3N2) specimens.

**Table 3:** Clade distribution and antigenic site mutations for influenza A(H3N2) viruses contributing to interim vaccine effectiveness evaluation, Canadian Sentinel Practitioner Surveillance Network, 1 November 2016–16 January 2017 (n = 221)^a^

Clade	Clade-defining amino acid substitutions (antigenic site)^b,c^	Distribution by province, % (column)									
		Alberta (n = 81)		BC (n = 81)		Ontario (n = 48)		Quebec (n = 11)		Total (n = 221)	
		n	%	n	%	n	%	n	%	n	%
**Clade 3C.2a**	N145S (A) + N144S (A) ( − CHO) + F159Y (B) + K160T (B) ( + CHO) + N225D (RBS) + Q311H (C)	0	0	0	0	0	0	0	0	0	0
**Clade 3C.2a variants**	Clade 3C.2a + Q197K (B) + R261Q (E)	0	0	1	1	0	0	0	0	1	0
	Clade 3C.2a + T131K (A) + R142K (A) + R261Q (E)	6	7	3	4	21	44	2	18	32	14
	Clade 3C.2a + N121K (D) + S144K (A) +/ − S219Y (D)	1	1	6	7	1	2	2	18	10	5
**3C.2a subtotal**		7	9	10	12	22	46	4	36	43	19
**Clade 3C.2a1**	Clade 3C.2a + N171K (D)	0	0	6	7	0	0	0	0	6	3
**Clade 3C.2a1 variants**	Clade 3C.2a + N171K (D) + N121K (D)	0	0	5	6	0	0	0	0	5	2
	Clade 3C.2a + N171K (D) + R142G (A)	0	0	1	1	0	0	1	9	2	1
	Clade 3C.2a + N171K (D) + N121K (D) + R142G (A)	9	11	23	28	10	21	0	0	42	19
	Clade 3C.2a + N171K (D) + N121K (D) + R142G (A) + I242V (D)	63	78	10	12	1	2	0	0	74	33
	Clade 3C.2a + N171K (D) + N121K (D) + T135K (A) ( − CHO) +/ − R142G (A) or T167S (D) or I242M (D)	2	2	23	28	6	13	2	18	33	15
	Clade 3C.2a + N171K (D) + N121K (D) + K92R (E) + H311Q (C) +/ − Q197R (B)	0	0	3	4	9	19	2	18	14	6
**3C.2a1 subtotal**		74	91	71	88	26	54	5	45	176	80
**Clade 3C.3a**	T128A (B) ( − CHO) + R142G (A) + N145S (A) + A138S (A) + F159S (B) + N225D (RBS)	0	0	0	0	0	0	2	18	2	1

BC: British Columbia; CHO: carbon-hydrogen-oxygen (glycosylation motif); RBS: receptor binding site.

^a^ Sequencing was attempted on all influenza A(H3N2) sentinel specimens contributing to VE analysis that had sufficient viral load and that were available up to 21 January 2017, with the last included collection date 16 January 2017. Genetic clade information was available for 221 of 263 (84%) viruses for which sequencing was attempted. Sequencing was not attempted on influenza A(H3N2) specimens with insufficient viral load (i.e. high CT value in the RT-PCR assay; n = 8) or those submitted after 21 January 2017 (n = 99).

^b^ Letters A through E refer to established antigenic sites in influenza A(H3N2) viruses [8,9]. RBS refers to the receptor binding site. Substitutions indicated with −CHO refer to mutations resulting in the loss of a potential glycosylation site; those indicated with +CHO refer to mutations resulting in the gain of a potential glycosylation site.

^c^ Additional substitutions in the egg-adapted high-growth reassortant vaccine strain are not considered here.

**Figure 2:**
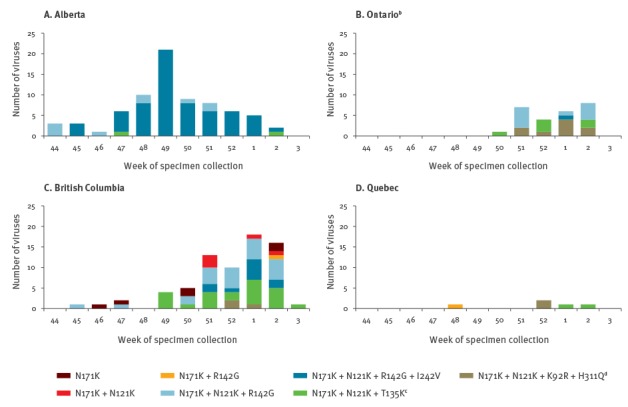
Distribution of clade 3C.2a1 variants by province^a^ and week of specimen collection, Canadian Sentinel Practitioner Surveillance Network (SPSN), 1 November 2016−16 January 2017 (n = 176)

^a^ Alberta and British Columbia are adjacent provinces located in western Canada; Ontario and Quebec are adjacent provinces located in central Canada, > 2,500 km away from Alberta and British Columbia.

^b^ Due to logistical issues, specimen collection did not begin until week 48 in Ontario.

^c^ +/− R142G or T167S or I242M mutations.

^d^ +/− Q197R mutation.

Considerable genetic heterogeneity was also observed among dominant but emerging clade 3C.2a1 variants by province and time (Figure 2).

In exploratory analyses, VE was highest and significantly protective in Alberta where an earlier epidemic start included a more limited range of clade 3C.2a1 variants dominated by N121K + R142G + I242V mutations (Figure 2, Table 2). Conversely, in the adjacent westernmost province of British Columbia and also further east in the provinces of Ontario and Quebec in central Canada, delayed epidemic activity was associated with lower VE and greater diversity in circulating clade 3C.2a1 variants, although confidence intervals overlapped for all four provinces.

## Discussion

Whereas the 2015/16 season was mild overall with late-season circulation of influenza A(H1N1)pdm09 viruses, the current 2016/17 season has been characterised to date by dominant influenza A(H3N2) activity, more comparable to the 2014/15 or 2012/13 seasons [1,10-12]. In the 2016/17 interim VE analysis reported here, we found overall vaccine protection of 42% (95% CI: 18–59) against medically-attended A(H3N2) illness, with variation by province that may reflect genetic heterogeneity in circulating A(H3N2) variants. This overall estimate is consistent with a recent meta-analysis of global studies based on the test-negative design that reported a pooled VE, including both interim and end-of-season estimates, of 33% (95% CI: 26–39) against seasonal A(H3N2) viruses [13]. Early VE estimates for the 2016/17 season available from Finland and Sweden found significant protection of 20–30% against laboratory-confirmed influenza in adults 65 years and older [14]; however, methodological details and influenza virus characterisations are not available for these estimates, limiting their interpretation.

Although still suboptimal given the substantial disease burden associated with influenza A(H3N2) seasons [15,16], our mid-season VE estimate for 2016/17 is considerably higher than the last A(H3N2)-dominated season in 2014/15 during which no vaccine protection was found [2,3]. In 2014/15, with unchanged vaccine components from the prior 2013/14 season and substantial antigenic drift in circulating viruses, negative interference from the prior season’s vaccination may have contributed to the historically low VE observed [3,17]. While more than 80% of vaccinated participants in 2016/17 were also vaccinated in the prior 2015/16 season (data not shown), higher VE than in 2014/15 was anticipated. This expectancy was in part based on the change in vaccine component from the prior 2015/16 season’s A/Switzerland/9715293/2013(H3N2)-like (clade 3C.3a) virus to the A/Hong Kong/4801/2014(H3N2)-like (clade 3C.2a) vaccine strain [18]. The latter is also considered a better antigenic match to circulating viruses than was the case in 2014/15 [18,19]. Specific evaluation of this hypothesis related to less pronounced effects of repeat vaccination for 2016/17 awaits end-of-season analyses. 

Circulating influenza A(H3N2) viruses in Canada and elsewhere this season have continued to evolve, with an increasing proportion since June 2015 clustering with the newly emerging clade 3C.2a1 that is distinguished by the HA1 substitution N171K, often combined with N121K, both in antigenic site D [20,21]. These clade 3C.2a1 variants are considered antigenically similar to the egg-adapted clade 3C.2a vaccine strain based on haemagglutination inhibition (HI) assay [1,19]. However, recent A(H3N2) viruses continue to be difficult to characterise antigenically by HI assay [20]. A potential glycosylation motif present at positions 158–160 in all clade 3C.2a and descendant viruses has resulted in variable agglutination of erythrocytes; loss or partial loss of this glycosylation motif during cell-culture passage may enable HI characterisation of a subset of clade 3C.2a viruses but also limit the generalisability of antigenicity findings on that basis [20,22].

In sequencing analysis, we identified considerable diversity among circulating influenza A(H3N2) strains, including a mix of genetic variants that differed geographically and with time. The majority (80%) of A(H3N2) viruses included in our VE analysis belonged to the newly emerging clade 3C.2a1, but with continuing genetic evolution compared with the vaccine strain. Almost all (95%) 3C.2a1 viruses had both the N171K and N121K mutations in site D that distinguish this clade. About two-thirds had acquired an additional R142G (site A) mutation, also present in all clade 3C.3 viruses and the majority of clade 3C.2a variants detected in this study, with or without an I242V mutation (site D). The clinical implications of accumulated antigenic site D mutations, representing a shift away from the heavily glycosylated but immunodominant sites A and B, requires further investigation [8,23]. Another 3C.2a1 variant, detected more frequently in the later study period but comprising only 15% of study viruses overall, had an additional T135K mutation in site A. T135K is associated with loss of a potential glycosylation site at positions 133–135 that has otherwise been present in all descendant A(H3N2) viruses since A/Sydney/5/1997 [24]. Changes in glycosylation motifs may be relevant to antigenicity, viral fitness and/or pathogenicity [24-26]. The ecological correlation between greater genetic diversity and lower VE by geographic region warrants further investigation in other countries, as well as end-of-season analyses.

Limitations of this analysis include the observational study design for which residual bias and confounding cannot be ruled out, and the small sample size resulting in wide confidence intervals, particularly in subgroup analyses. Although interim estimates are generally considered a reliable predictor of final estimates, this reliability depends in part upon the stage of the epidemic and virus evolution, and contributing virological and participant profiles, at the time of the mid- and end-of-season analyses [27]. Of particular note, Alberta had an earlier start to the influenza season and findings may not reflect the full diversity or distribution of evolved variants or VE estimates for the remainder of the season. Given the high specificity of RT-PCR assays for influenza viruses, differences in diagnostic test characteristics between provinces are unlikely to have influenced VE findings [28]. VE estimates are subject to change and are provided here only for influenza A(H3N2); if feasible, VE against other types/subtypes, as well as clade-specific VE, will be explored and compared with findings from other settings in end-of-season analyses.

## Conclusion

We report interim VE of ca 40% for the 2016/17 influenza A(H3N2) epidemic in Canada, which is higher than in 2014/15 and consistent with expected but suboptimal VE estimates for influenza A(H3N2) more generally. Given that a substantial proportion of vaccinated people may remain unprotected against influenza A(H3N2) illness, other adjunct measures should be considered to minimise associated morbidity and mortality, particularly among high-risk individuals. Continued evolution in circulating 3C.2a variants and their derivatives, and the impact on vaccine protection, warrants ongoing monitoring to inform potential vaccine reformulation.

## GenBank Accession Numbers

KY583507 – KY583727.
